# Supplementary material for: A consensus-based decision model for assessing the health systems
Source: PLoS One. 2020 Aug 18;15(8):e0237892. doi: 10.1371/journal.pone.0237892 (PMC7437456; doi:10.1371/journal.pone.0237892)
Supplement: Attachment — Submitted filename: Response to reviewer 2.docx [file pone.0237892.s002.docx]

Reviewer #2: The paper analyses an actual topic and it is interesting to potential readers. The paper is well prepared, it contains all required parts of a scientific paper.

I have only one critical comment. References are up to 2016. Therefore the authors should update the literature review.

Many thanks for your valuable comments. We have added several references after 2016 in Section 1.
